# Case report on the diagnosis of vascular thoracic outlet syndrome followed by mechanical thrombectomy

**DOI:** 10.1016/j.ijscr.2023.109019

**Published:** 2023-11-04

**Authors:** Emma Karina Martínez-Cárdenas, Adrian Torres-Parlange, Jorge Sotelo-Carbajal, René Edivaldo Hernández-Zamora, Arnold García-Ledezma, Quitzia Libertad Torres-Salazar

**Affiliations:** a Hospital General "5 de Diciembre" of the Security Institute for the Service of State Workers, México; b Hospital General Regional No. 1 Tijuana, Baja California, Mexican Institute of Social Security, México; c Universidad Juárez del Estado de Durango, México

**Keywords:** Case report, Venous thrombosis, Thoracic outlet, D-dimer, Venous thoracic outlet syndrome

## Abstract

**Introduction and importance:**

The thoracic outlet syndrome is characterized by compression of the brachial plexus or subclavian vessels due to anatomical alterations of the thoracic cavity. Vascular presentation is rare and includes thromboembolism and edema in the upper limb, and the diagnosis is often elusive due to its rarity. In this case, we describe a vascular thoracic outlet syndrome presentation whose diagnosis through angiography was achieved after a mechanical thrombectomy.

**Case presentation:**

We report a 43-year-old female patient with pain in the right upper limb, accompanied by edema and mild violet discoloration, without risk factors for hypercoagulability, with D-dimer levels within normal values. Mechanical thrombectomy with AngioJET was performed via an endovascular approach, with the extraction of multiple clots, confirming the presence of thoracic outlet syndrome as the underlying cause of the current condition.

**Clinical discussion and conclusions:**

Venous thoracic outlet syndrome is a challenging entity to diagnose; however, it should be considered in cases of deep vein thrombosis of the subclavian vein and confirmed by angiography after a thrombectomy.

## Highlights

• We describe a vascular thoracic outlet syndrome presentation
• Mechanical thrombectomy using AngioJET revealed thoracic outlet syndrome as the root of her symptoms.
• This condition can manifest as deep vein thrombosis in the subclavian vein, and its rarity makes it difficult to diagnose.

## 1 Introduction

Thoracic outlet syndrome (TOS) is a pathology of several etiologies and clinical manifestations with a common characteristic, the compression of one or more elements that cross the thoracic outlet in question [1]. There are two main etiologies that comprise the thoracic outlet: Nervous or neurogenic and vascular (arterial or venous) [2]. The neurogenic presentation is the most common, and vascular presentation occurrs in <10 % of cases [3]. Vascular thoracic outlet syndrome (vTOS) can be primary (usually congenital) or secondary (often due to thrombosis or stenosis). Secondary vTOS is commonly caused by external factors like tumors, or intrinsic factors like coagulopathies [4]. The patient received care in a public healthcare hospital (Hospital General ISSSTE 5 de diciembre) in Mexicali, a city of Baja California Norte in Mexico.

## 2 Presentation of case

Female patient, aged 43, originally from Sinaloa in the northwest of Mexico, currently residing in Jalisco in the west of Mexico, presented with a significant history of Gentamicin allergy and underwent laparoscopic cholecystectomy in 2018. Chronic-degenerative diseases, transfusions, smoking, alcoholism, and drug addiction were denied.

In February 2023, she complained of pain in the right upper limb, accompanied by edema and slight purplish discoloration. She was admitted to the emergency department of the public regional hospital in Jalisco, where a complete blood count, blood chemistry, liver and kidney profile, coagulation times, and D-Dimer were performed, revealing values within the normal range (282 ng/ml). She was discharged with an improved condition, managed with analgesics at home, and given an open appointment for further evaluation.

In the following days, she experienced an increase in the volume of the right upper extremity and difficulty mobilizing the fingers, leading her to seek care at the emergency department. A new D-Dimer measurement was taken, showing a result of 560 ng/ml. She was discharged with analgesics and an ordinary request for Doppler ultrasonography.

In March, during her visit to the city of Mexicali, she presented discoloration with edema in the right extremity and received treatment at the General Hospital. Comprehensive laboratory tests were conducted, all showing normal results except for a D-Dimer value of 1100 ng/ml. Computed Axial Tomography (CT) revealed findings compatible with deep vein thrombosis of the right subclavian, axillary, and brachial veins, as well as thrombophlebitis of the right basilic vein. A diagnosis of atypical deep vein thrombosis of the subclavian, axillary, and right brachial veins was made. She was admitted to the hospital and commenced on a regimen of subcutaneous Enoxaparin 60 mg every 12 h and nonsteroidal anti-inflammatory drug (NSAID)-based analgesics.

During her hospitalization, she showed significant improvement in symptoms. Suspecting Antiphospholipid Syndrome or an autoimmune disease, IgA, IgG, and IgM anticardiolipin antibodies, and antinuclear antibodies were tested via the ELISA method, all yielding normal results.

**Fig. 1:**
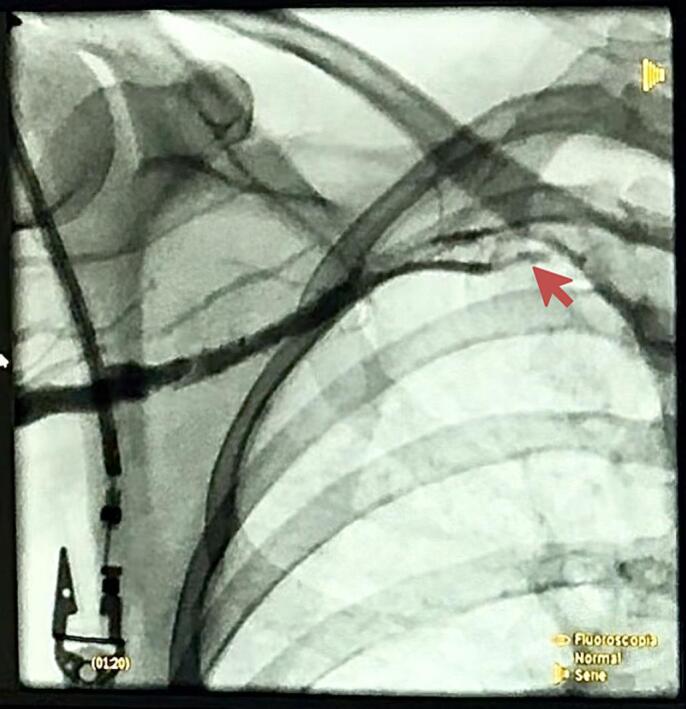
Angiography of the right subclavian vein in abduction with evidence of compression of the subclavian vein secondary to the thoracic outlet.

The patient received integrated management from vascular and thoracic surgery. A mechanical thrombectomy with AngioJET was scheduled and performed via an endovascular approach through the right brachial artery guided by ultrasound. A 6Fr introducer was inserted, and a 260 × 0.035 cm hydrophilic guidewire was advanced to the superior vena cava, revealing complete occlusion of the right basilic, axillary, and subclavian veins. The AngioJET Omni catheter was then utilized for 240 s in 4 cycles of 60 s each, successfully restoring permeability, as confirmed by follow-up phlebography. Additionally, the presence of a thoracic outlet was identified as the underlying cause of the current condition (Fig. 1).

During the initial postoperative hours, there was a slight elevation in BUN levels while creatinine remained within the normal range. Consequently, fluid replacement therapy was administered. Two days later, she was recommended for discharge due to notable clinical improvement. The patient was re-evaluated one week after the vascular surgery, showing a decrease in symptoms. However, signs of post-thrombotic syndrome persisted, including mild edema in the affected limb and diminishing pain, which had been gradually decreasing since her hospital discharge. An appointment in the Cardiothoracic Surgery service was scheduled one month after the intervention, during which a conservative approach based on the administration of oral anticoagulants was chosen. A subsequent appointment in the Vascular Surgery service was arranged for 3 months post-surgery, but the patient failed to attend the scheduled appointment. There has been no further follow-up since the last consultation with the Cardiothoracic Surgery service, likely due to the patient's residence in another state (Jalisco, Mexico). The work has been reported in line with the SCARE criteria [5].

## 3 Discussion

TOS is a complex condition involving the compression of the brachial plexus or subclavian vessels due to anatomical changes in the thoracic cavity, leading to neurogenic and/or vascular symptoms [1,6,7]. It typically occurs in three spaces (scalene triangle, costoclavicular space, subcoracoid) and can have congenital, acquired, or traumatic causes [8].

TOS is generally viewed as a single condition, but it can be categorized into three types based on the structure that is compressed: 1. Neurogenic TOS, involving compression of the brachial plexus from C5-T1 nerve roots, 2. Vascular compression, which can affect arteries or veins, and 3. Non-specific TOS, indicating potential brachial plexus involvement [9]. Another classification distinguishes between two primary etiologies within the thoracic outlet: Neurogenic and vascular TOS (arterial or venous) [2].

The neurogenic type represents about 95 % of cases in patients and is the primary focus of clinical evaluation due to its prevalence. Distinguishing between muscle pain and neurogenic pain can be difficult due to their close correlation [[10], [11], [12]]. TOS is rare in female patients and according to the American College of Rheumatology, the incidence in the United States of neurogenic and vTOS is estimated to be 25 and 8 cases per 1,000,000 people per year, respectively [2].

This case report describes an atypical clinical case of >6 weeks of evolution in a middle-aged female patient attributed to vTOS. vTOS can have either a primary (congenital or idiopathic) or secondary (stenosis or thrombosis) origin. In primary cases, the cause is uncertain but is believed to involve congenital narrowing of the space where the subclavian vein meets the innominate vein. Secondary vTOS, as discussed here, is often linked to thrombosis or stenosis, primarily caused by medical procedures like central venous line placement or medical guide introductions. It can also result from external factors like tumors or internal factors such as coagulopathies [4,13]. The probable cause of thrombosis in this case is the thoracic operculum due to a probable malformation of the first rib, which was observed in the confirmatory image presented, visualizing the interruption of blood flow with contrast when performing abduction of the affected thoracic limb.

The clinical presentation of vTOS varies among patients. Common symptoms include increased volume and a sense of heaviness in the affected upper extremity, along with moderate pain or even asymptomatic cases [8,14]. In acute vTOS cases, symptoms are pronounced and include diffuse edema, cyanosis, pain, and palpable thrombus in the veins. Chronic vTOS can lead to collateral venous pathways and venous dilation in the cervical region, upper thorax, and shoulders [1,10,15]. The patient described in this report presents pain in the right upper limb, accompanied by edema and slight purplish discoloration, indicating an acute occlusion.

Patients with vTOS can be categorized into three groups depending on their symptoms. The first group experiences position-dependent compression without thrombosis, causing symptoms during specific upper limb movements (known as McCleery Syndrome) [12]. The second group has intrinsic subclavian vein stenosis due to factors like scalene muscle hypertrophy and friction with the first rib, leading to fibrosis and stenosis. The third group presents occlusive thrombosis, often associated with strenuous physical activity, referred to as Paget-Schroetter syndrome or stress thrombosis [1,14,16]. However, some cases of upper limb venous thrombosis lack a history of strenuous activity or anatomical issues and are considered idiopathic [15]. In these cases, hypercoagulable states, such as contraceptive use or medications, may contribute to vTOS symptoms, but this was not the case for the patient described.

The diagnosis of vTOS involves assessing the patient's clinical history, conducting a thorough physical examination, and utilizing imaging studies such as computed tomography, Doppler ultrasound of the affected limb, or venogram [3]. A venogram entails injecting contrast directly into the vein under study, with possible thrombolysis if needed. However, diagnosing vTOS can be challenging due to its rarity [9,16]. In this case, the definitive diagnosis was based on angiography findings and symptoms, even though the expected D-dimer elevation was absent. Differential diagnoses considered included C4-C5 cervical disc herniation, painful shoulder syndrome, lymphedema, and unspecified muscle injury. While D-dimer is highly specific for thrombotic conditions, it did not exhibit the anticipated high value for its Negative Predictive Value (NPV), suggesting a lower likelihood of vTOS. Notably, there is no established concentration value for vTOS in the literature due to its diverse presentations.

The initial treatment for suspected vTOS involves intravenous low molecular weight heparin to stabilize and prevent thrombus extension. Thrombus management varies with thrombus age: recent thrombi (<2 months) are treated with thrombolysis, with a success rate of 80–99 %, albeit with a small risk (1–12 %) of bleeding complications [14,17]. Pharmacomechanical thrombectomy is effective. If the vein remains unoccluded with minimal or no stenosis, elective first rib resection with subclavian extrinsic venolysis is advised [17]. In the present case, mechanical thrombectomy with AngioJET was performed, however, referral to Thoracic Surgery is made for definitive management of the thoracic operculum and follow-up with vascular surgery.

vTOS is an entity with a complicated diagnosis, however, mechanical thrombectomy can be helpful to support the diagnosis, in addition to being considered a part of the treatment. Although the D-dimer does not initially show alarm signs, it should be monitored as the patient does not present a favorable response to the symptoms presented.

## Consent

The patient's written consent is obtained for the publication of data and photographs.

## Ethical approval

This study does not require committee approval because it is a case report; however, we have the patient's authorization to use this material for publication.

## Funding

Nothing to declare.

## Author contribution

EKMC - Diagnosis and follow-up

ATP- Surgical approach plan

JSC- Surgical assistant

REHZ- File tracking and documentation

AGL- Bibliographic review

QLTS- Article redaction

## Guarantor

Quitzia Libertad Torres Salazar

## Conflict of interest statement

Nothing to declare.
